# PLANEX: the plant co-expression database

**DOI:** 10.1186/1471-2229-13-83

**Published:** 2013-05-20

**Authors:** Won Cheol Yim, Yongbin Yu, Kitae Song, Cheol Seong Jang, Byung-Moo Lee

**Affiliations:** 1 Department of Plant Biotechnology, Dongguk Univ-Seoul, Seoul, 100-715, Korea; 2 Department of Healthcare informatics, The Catholic University of Korea, Seoul, 137-701, Korea; 3 Department of Applied Plant Sciences, Kangwon National University, Chuncheon, 200-701, Korea

**Keywords:** Co-expression, Database, Pearson’s correlation coefficients, Clustering

## Abstract

**Background:**

The PLAnt co-EXpression database (PLANEX) is a new internet-based database for plant gene analysis. PLANEX (http://planex.plantbioinformatics.org) contains publicly available GeneChip data obtained from the Gene Expression Omnibus (GEO) of the National Center for Biotechnology Information (NCBI). PLANEX is a genome-wide co-expression database, which allows for the functional identification of genes from a wide variety of experimental designs. It can be used for the characterization of genes for functional identification and analysis of a gene’s dependency among other genes. Gene co-expression databases have been developed for other species, but gene co-expression information for plants is currently limited.

**Description:**

We constructed PLANEX as a list of co-expressed genes and functional annotations for *Arabidopsis thaliana*, *Glycine max*, *Hordeum vulgare, Oryza sativa*, *Solanum lycopersicum, Triticum aestivum, Vitis vinifera* and *Zea mays.* PLANEX reports Pearson’s correlation coefficients (PCCs; *r*-values) that distribute from a gene of interest for a given microarray platform set corresponding to a particular organism. To support PCCs, PLANEX performs an enrichment test of Gene Ontology terms and Cohen’s Kappa value to compare functional similarity for all genes in the co-expression database. PLANEX draws a cluster network with co-expressed genes, which is estimated using the *k*-mean method. To construct PLANEX, a variety of datasets were interpreted by the IBM supercomputer Advanced Interactive eXecutive (AIX) in a supercomputing center.

**Conclusion:**

PLANEX provides a correlation database, a cluster network and an interpretation of enrichment test results for eight plant species. A typical co-expressed gene generates lists of co-expression data that contain hundreds of genes of interest for enrichment analysis. Also, co-expressed genes can be identified and cataloged in terms of comparative genomics by using the ‘Co-expression gene compare’ feature. This type of analysis will help interpret experimental data and determine whether there is a common term among genes of interest.

## Background

A combination of methodologies from the fields of genomics, proteomics and bioinformatics provides a powerful approach to investigating biological processes. Biological functions of genes are usually determined by the interaction of a protein or gene product, and gene expressions are frequently related in biological processes. Therefore, co-expressed genes might be related in a biological pathway and may provide information critical for understanding complex biological systems [1,2]. Many technical approaches have been used in genome-wide experiments, and the ability to measure the regulation of several thousand genes simultaneously has revolutionized the way biological processes are analyzed. To understand biological systems, co-expression data have been used in a wide variety of experimental designs, including gene targeting, regulatory investigations and identification of potential partners in protein-protein interactions [3].

Substantial amounts of such expression data are required to estimate co-expressed gene dependency. Unfortunately, these experiments are costly and time consuming. However, a vast number of gene expression data sets have recently become available for several plant species. The most popular public microarray databases are ArrayExpress [4], Gene Expression Omnibus (GEO) [5], NASCArrays [6] and Genevestigator [7]. Still, it is difficult for biological researchers to manage this large amount of gene expression data without a background in bioinformatics. To this end, the field of bioinformatics has accelerated co-expression analysis of biological processes. In addition, the completion of the genome sequences of the model plants *Arabidopsis thaliana*[8], *Glycine max*[9], *Oryza sativa*[10], *Solanum lycopersicum*[11]*, Vitis vinifera*[12] and *Zea mays*[13] have advanced genome and gene expression analysis. For other species with poorly resolved gene expression data, such as *Hordeum vulgare* and *Triticum aestivum*, genome resources are improving with The Gene Index Project by the Dana Faber Cancer Institute (DFCI) [14]. The annotated genome sequences have stimulated the development of a number of functional genomic approaches. These materials are valuable for gene expression in genome-scale microarrays.

During the co-expression data set construction, the gene expression data were normalized with summarization methods, including RMA [15], GCRMA [16] and MAS5 [17]. One method of identifying co-expressed gene sets is through the estimation of gene expression similarity. The most convenient way to estimate gene expression similarities is to use Pearson's correlation coefficients (PCCs) [1,18]. If similarity is determined by a correlation metric (e.g. PCCs), a comprehensive pairwise matrix of correlation values are generated that represents expression similarity.

Based on co-expression data set analysis, we focused on improving the construction of gene networks. Principal components analysis (PCA) is a popular technique used to find the major component of a multivariate dataset. In DNA microarray analysis, it is used to find the gene groups that cooperatively change expressions over several experiments [19], and PCA is done in gene space. Then, the *k*-mean cluster algorithm is combined to reveal samples with large contributions.

Plant co-expression databases have previously been constructed for *Arabidopsis thaliana*, *Oryza sativa* and *Hordeum vulgare.* These databases, the Arabidopsis Co-expression Toolkit (ACT) [20], STARNET 2 [21], RiceArrayNet [22], ATTED-II [23], Co-expressed biological Processes (CoP) database [24] and PlaNet [25], are used for searching co-expression relationships and incorporating functional data. Given the recent rapid growth of high performance computers with the ability to perform rapid calculations, co-expression database construction is possible using large-scale gene expression data.

In this report, we describe the construction and use of the PLAnt co-EXpression database (PLANEX; Additional file 1: Table S1) and discuss the output produced by user query. PLANEX mines already-computed gene pair correlations across eight species of plants. With PLANEX, we provide *Arabidopsis thaliana, Glycine max, Hordeum vulgare, Oryza sativa, Solanum lycopersicum, Triticum aestivum, Vitis vinifera* and *Zea mays* co-expression data sets with a user-friendly web interface for retrieving co-expressed gene lists and functional enrichment data of interest. A central motivation for constructing PLANEX was to leverage massive resources of microarray data for biological interactions, expression diversity and the discovery of putative gene regulatory relationships prior to conducting additional costly wet lab experiments. This database provides details that may aid in understanding expression similarity and functional enrichment of input genes.

## Construction and content

### Expression data

Raw microarray data were obtained from the GEO of the National Center for Biotechnology Information (NCBI) through April 2011. We selected data from *Arabidopsis thaliana, Glycine max, Hordeum vulgare, Oryza sativa, Solanum lycopersicum, Triticum aestivum, Vitis vinifera* and *Zea mays* Affymetrix GeneChip Genome Array, which is one of the most frequently-used and publicly-deposited platforms for plants (Table 1).

**Table 1:** Co-expression data information contained in PLANEX

**Species**	**Affymetrix GeneChip**	**Number of microarray slides**	**Micorarray platform**	**Source database of coding sequence**
*Arabidopsis thaliana*	ATH1	5502	GPL198	Phytozome^1^
*Glycine max*	Soybean	3080	GPL4592	Phytozome
*Hordeum vulgare*	Barley1	738	GPL1340	DFCI^2^
*Oryza sativa*	Rice	884	GPL2025	Phytozome
*Solanum lycopersicum*	Tomato	253	GPL4741	Phytozome
*Triticum aestivum*	Wheat	451	GPL3802	DFCI
*Vitis vinifera*	Vitis vinifera	738	GPL1320	Phytozome
*Zea mays*	Maize	379	GPL4032	Phytozome

^1^Dana Faber Cancer Institute (DFCI), http://compbio.dfci.harvard.edu/tgi/.

^2^Phytozome v 9.0, http://www.phytozome.net.

All of the raw data (in CEL file format) were downloaded through programmatic access to GEO ( http://www.ncbi.nlm.nih.gov/geo/info/geo_paccess.html). We terminated GEO Series (GSEs) that included truncated GEO Sample (GSM). The cross platform GSMs were also terminated, including GSE13641 (*Rorippa amphibia* expression profile on *Arabidopsis thaliana* Affymetrix GeneChip platform; GPL198). We also collected raw data, with the exclusion of subspecies expression data, including *Glycine soja* on the *Glycine max* platform (GPL4592; e.g. GSE20323) and *Arabidopsis lyrata subsp. petraea* and *Arabidopsis halleri* on the *Arabidopsis thaliana* Affymetrix GeneChip platform (GPL198; e.g. GSE5738).

The CEL files were used for summarizing probe sets, which were the results of the intensity calculations on the chip pixel value. All expression levels were analyzed using background subtraction, normalization and summarizing probe sets. We estimated quantile normalization using an RMA algorithm for detecting the background information. All microarrays were computed probe sets that summarized each of the eight species using Affymetrix Power Tools [26].

### Implementation

**Figure 1:**
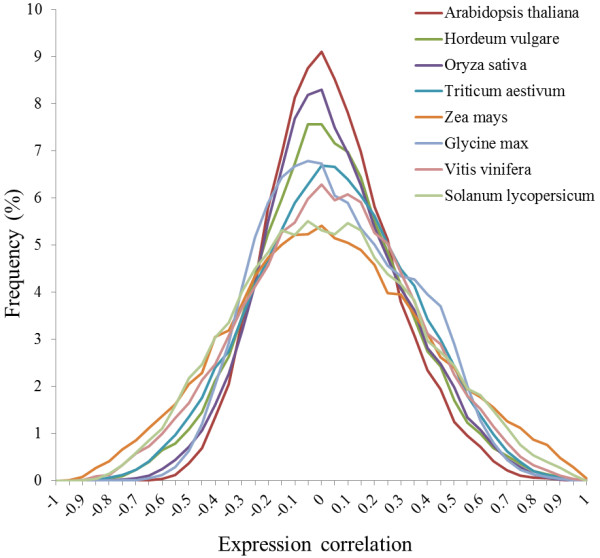
Frequency distribution of PCCs of randomly selected gene pairs.

The gene co-expression data were entered in the PLANEX system by pre-implementation. The data were implemented with expression probe set summarizing data. We provided PCCs to assess the extent of gene co-expression, and we developed novel C++ codes to generate co-expression data. The pairwise co-expression calculations did not require heavy CPU power, but numerous CPUs helped reduce calculation time. We used the GAIA system at the Supercomputing Center of the Korea Institute of Science and Technology Information, [27] which contained 1536 CPU cores. The GAIA system is based on Advanced Interactive eXecutive (AIX) by IBM, which supports Message Passing Interface (MPI) [28]. Our unique C++ code supported MPI and co-expression data were estimated by 512 CPU cores. To retrieve co-expression data, we set thresholds for co-expression values. To specify positive (top 1% of PCCs) and negative (bottom 1% PCCs) values for co-expressed gene sets, the distribution of random gene pairs was assessed by PCCs (Figure 1). The number of random gene pairs corresponded to the number of probes on the array (Table 2).

**Table 2:** The thresholds for co-expression values

**Species**	**No. of probes**	**Positive**^**1**^	**Negative**^**2**^
*Arabidopsis thaliana*	22,810	0.585	−0.465
*Hordeum vulgare*	22,840	0.68	−0.625
*Oryza sativa*	57,381	0.646	−0.535
*Triticum aestivum*	61,290	0.686	−0.635
*Zea mays*	17,734	0.835	−0.775
*Glycine max*	61,170	0.645	−0.505
*Vitis vinifera*	16,602	0.715	−0.715
*Solanum lycopersicum*	10,209	0.775	−0.705

^1^ Positive indicated PCCs ≤ 0.01.

^2^ Negative indicated PCCs ≥ 0.99.

### Clustering

For clustering, the gene expression values were used for analysis. We applied the *k*-mean clustering method to the expression data, which assigned each point to the cluster whose center was nearest [29]. We used the PCA to determine the number of cluster *k*. The PCA was conducted using CLUSTER, so that the clusters were ordered and chosen to maximally explain the remaining variance in data vectors [1]. Consequently, the *k*-mean clusters were analyzed with the number of clusters in each species. The large amount of expression data required long-term clustering time. Therefore, we compiled the Parallel K-mean Data Clustering code [30], which was executed on the AIX supercomputer system with MPI. The *k*-mean algorithm provided nodes of the co-expression network in PLANEX.

### Mapping gene identifiers onto probe set IDs

The genome sequence and annotation project Phytozome was recently completed and released [31]. We clarified annotations and sequences of the species by downloading all Affymetrix GeneChip probe sequences [26], and we mapped them against the probe to the nucleotide of the genome of six sequenced plants: *Arabidopsis thaliana, Glycine max, Oryza sativa, Vitis vinifera,Solanum lycopersicum* and *Zea mays (*Phytozome V9.0). In contrast, other species whose genome sequences are still unfinished, such as *Hordeum vulgare* and *Triticum aestivum*, were mapped with Tentative Consensus sequences from DFCI. The probe matches were made using our unique Perl script. The script processed string-matched nucleotide sequences (including reverse complement) against an individual GeneChip probe of any given species and returned a list of probe set affinities that corresponded to the sequence of each species. Specifically, *Zea mays* had 15 sequence pairs per probe, and all other plant species had 11 pairs per probe.

### Gene ontology term assignment

Due to the hierarchical tree of the gene ontology (GO) terms and redundancy of the terms, we mapped GO terms against representative gene function. The DFCI provided GO mapping annotation. Phytozome sequence annotation did not support GO mapping annotation, but it did provide Pfam IDs; we mapped the representative Pfam IDs against GO terms. We mapped the external classification system to GO [32]. GO-TermFinder was used to estimate the enrichment of GO terms [33]. GO-TermFinder was integrated into PLANEX using a web interface, which evaluated the enrichment of the principle GO categories, including cellular components, biological processes, and molecular functions with hypergeometric distribution and a False Discovery Rate (FDR) described by Benjamini and Hochberg.

### Comparative analysis of co-expressed gene sets

Cohen’s Kappa statistics were used to compare co-expression data between species [34]. An in-house module similar to the online DAVID tool [35,36] was used to evaluate co-expression similarity using Kappa statistics, which were integrated using a web interface. A protein sequence was used to select two genes from among the species *Arabidopsis thaliana*, *Glycine max*, *Oryza sativa*, *Vitis vinifera* and *Zea mays*. After two query genes were submitted, the module compared the co-expression data set of each query gene, which were converted to the Pfam ID [37]. The Kappa measured the percentage of data values in the main diagonal of the table and then adjusted those values for the amount of agreements that could be expected due to chance alone.

### System development

The web application of PLANEX was developed with Dancer (Perl web application framework) [38] for the server side and JQuery (Javascript framework) [39] for the client side. The co-expression database was combined with MongoDB (document-oriented database) [40] and TokyoCabinet (management of database) [41]. MongoDB stored co-expression data as a document file, making the integration of data in pairwise co-expression applications easier and faster. TokyoCabinet stored gene ID data by a single key and used hashing techniques to enable fast retrieval of co-expression data of the query gene. This combination markedly improved the processing and accessing speeds of searches. We used the Cytoscape Web [42] to display the network on internet browsers. The Cytoscape Web does not require the installation of a plugin and works fast for all kinds of browsers. PLANEX operates on a Ubuntu 10.04 [43] sever equipped with a 2.66GHz dual CPU and 8GB RAM.

## Utility and discussion

### Web interface

**Figure 2:**
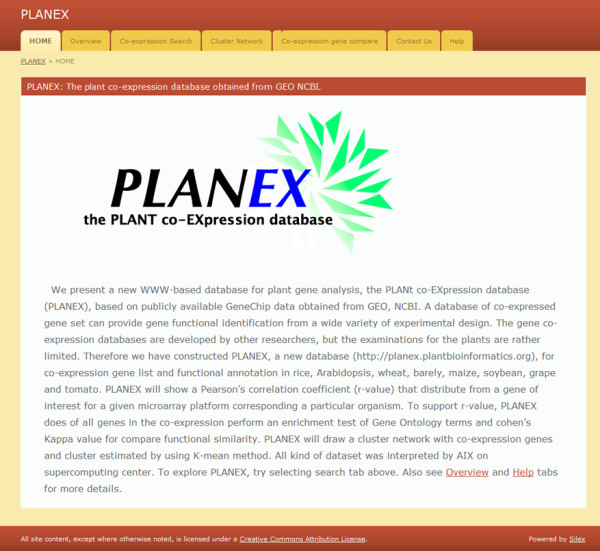
The homepage of PLANEX.

**Figure 3:**
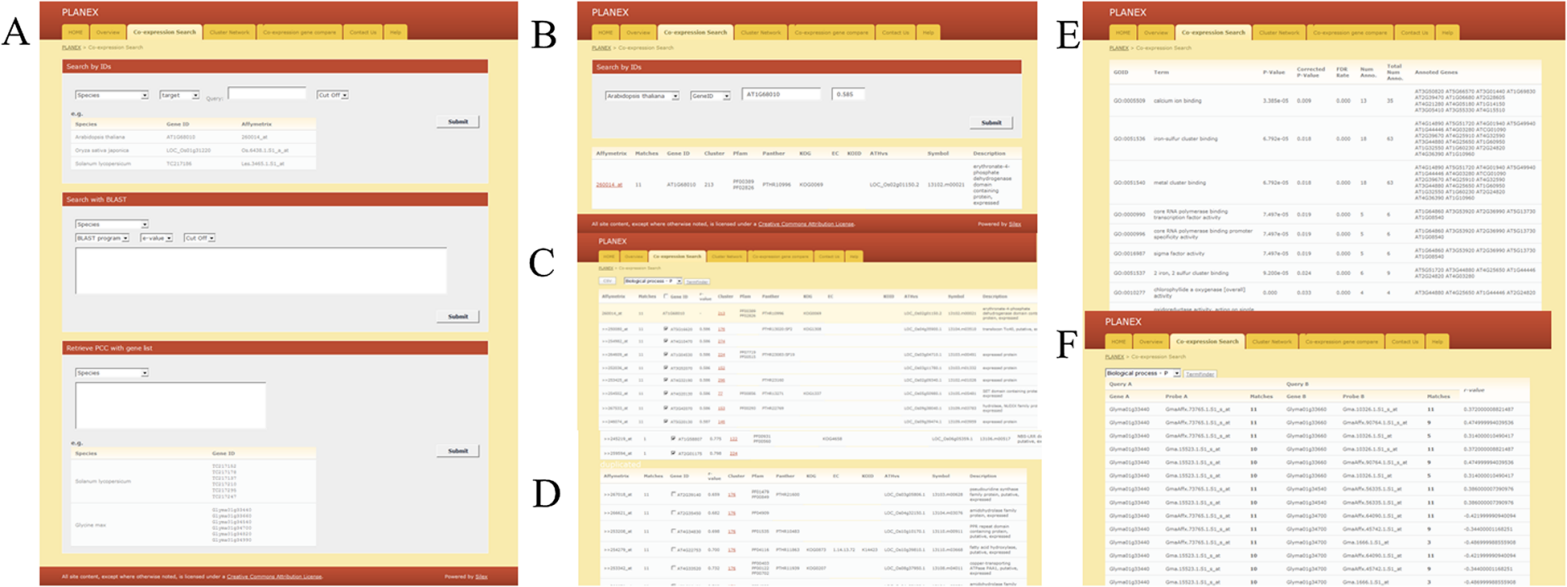
**An example of a ‘Co-expression Search’.** (**A**) The layout of ‘Co-expression Search’. (**B**) The results of probe mapping. (**C**) Co-expression search result. (**D**) Co-expressed genes with duplicated probe or gene redundancy. (**E**) Results of a statistical test for gene functional enrichment. (**F**) Search result of ‘Retrieve PCCs with gene list’.

PLANEX can be accessed through a user-friendly web interface ( http://planex.plantbioinformatics.org/, see Avaliability an requirements section) that provides three search menus: ‘Co-expression search’, ‘Cluster network’, and ‘Co-expression gene compare’ (Figure 2). The ‘Co-expression search’ can be used for co-expressed gene sets and PCC values. To search the database, an Affymetrix GeneChip ID or a representative gene ID is used to ‘Search by IDs’ or a paste sequence is used to ‘Search with BLAST’ [44]; two or more representative gene IDs are used to ‘Retrieve PCC with gene list’ (Figure 3A). As shown in Figure 3A, PLANEX depends on the selection of options such as species, target, cut-off, BLAST program and e-value. The distributions of random genes were determined to be cut-off values in each species.

After a query is submitted to ‘Search by IDs’ or ‘Search with BLAST’, the probe match results page is shown. The probe match page indicates the number of probes matching the query over the total number of probes, as well as their affinity, shown as ‘match’ (Figure 3B). This probe match page will help discard redundant probes to genes. PLANEX finds many co-expressed genes within the cut-off values (Figure 3C). The duplicated Affymetrix IDs are indicated in the ‘Duplicated’ section of the results page (Figure 3D). The co-expressed gene set can be downloaded in CSV format for analysis by GO-TermFinder. GO-TermFinder provides three GO term enrichment analyses with a hypergeometric *p*-value < 0.05 at FDR ≤ 10–6 (Figure 3E). After submitting a query to ‘Retrieve PCCs with gene list’, the gene list will show the correlation in pairwise format (Figure 3F). PLANEX does not provide a probe match page, but, instead, it provides all potentially matching probe sets for a gene list, which indicate PCCs and affinity. The data are supported by GO-TermFinder, which is similar to the other searches.

**Figure 4:**
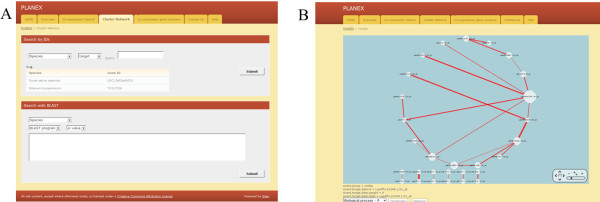
**An example of ‘Cluster Network’.** (**A**) The layout of ‘Cluster Network’. (**B**) The query genes in the co-expressed gene network.

PLANEX allows co-expression network data to be displayed in a browser. The ‘Cluster network’ is based on *k*-mean cluster analysis and PCCs, which support ‘Search by IDs’ and ‘Search with BLAST’ functions (Figure 4A). The network consists of the results of the *k*-mean cluster analysis, indicated as node, size of node, represented number of the edge, and the edge indicated by PCCs (Figure 4B).

**Figure 5:**
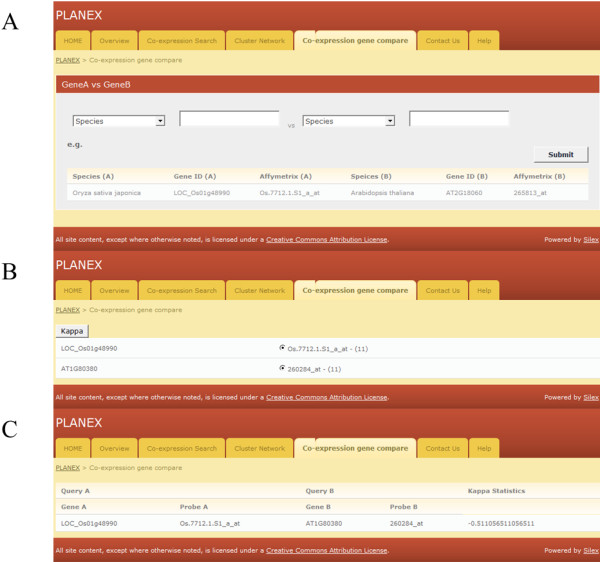
**An example of ‘Co-expression gene compare’.** (**A**) The layout of ‘Co-expression gene compare’. (**B**) The results of probe mapping. (**C**) Results of a Cohen’s Kappa statistical test for the query genes.

The Kappa statistics analysis tools in PLANEX can be used to compare co-expressed genes with other species, using the ‘Co-expression gene compare’ feature (Figure 5). It accepts only *Arabidopsis thaliana, Glycine max, Oryza sativa, Vitis vinifera* and *Zea mays* as protein annotated plant gene IDs. Any two species can be compared with their representative gene ID from Phytozome. The simple Kappa statistics coefficients show the agreement between two co-expressed gene sets, which is measured on a binary scale. This analysis is useful in comparative genomics to determine the similarity of co-expressed gene sets or the functional similarity of family genes. This approach provides a comparative analysis with commonly reported measurements in the medical literature.

## Discussion

PLANEX is a novel database that helps researchers study complex biological processes by co-expressed gene sets overlayed onto a *k*-mean cluster. ATTED-II, STARNET 2, RiceArrayNet and CoP provide co-expression relationships, but they contain only one to three sets of co-expression data. Therefore, an advantage of PLANEX is that it combines sets of co-expression data from eight different species. Additionally, it clusters and compares members of co-expressed genes. As far as we know, PLANEX is the only system that combines cluster and PCCs data.

Another advantage of PLANEX is that probes were mapped against representative genes by string match instead of BLAST. Our probe match script produced positive results if each base in a probe sequence matched perfectly with the representative gene sequence without any gap.

One potential application in PLANEX is GO-TermFinder. We generated a *Saccharomyces* Genome Database (SGD) file format for each species. Model species like *Arabidopsis thaliana* and *Oryza sativa* have a large set of functionally annotated genes with GO terms supported by various experimentally-derived evidence codes. In contrast, other organisms only have annotations inferred through electronic annotation (e.g., *Vitis vinifera* and *Zea mays*) or completely lack functional annotation. Since we initially lacked functional GO data, we converted Pfam to GO IDs and built an SGD file for functional enrichment analysis. However, this mapping should be used only as a guide.

Our previous report of *Oryza sativa* genome duplication [45] evidenced the positive (top 1% of PCCs) value as 0.545, but we used 0.646 as the positive PCCs threshold in *Oryza sativa* for this report. We established this different criterion because we included more than the given number of microarrays, since we believed that more microarrays generated more significance for the expression study. Also, Aoki et al. [46] specified a minimum PCCs value (0.55-0.66) for co-expressed gene retrieval to minimize false gene function relationships. We provided a particular threshold to retrieve co-expressed genes for each species that showed normal distribution (Figure 1).

The ‘Co-expression gene compare’ tab on the PLANEX menu provides data for comparative genomics. The *Arabidopsis* genome is believed to contain similar gene numbers to the rice genome, and both have undergone a whole genome duplication event [47,48]. The use of Kappa statistics coefficients is expected to be in accordance with the degree of expression divergence of the data. Previously, we reported that the rice gene families evidenced a similar high degree of expression diversity between members using rice public microarrays [45]. The comparison of co-expressed genes may support the understanding of specialization in the direction of complex biological processes between members of a gene family over evolutionary time [49].

## Conclusions

The small, but important, function of comparing co-expressed genes may provide clues to the molecular functional conservation or diversity between orthologus genes, particularly *Poaceae* family genes. PLANEX can be used to interpret results of co-expressed genes and, also, to perform delicate analyses in comparative genomics. PLANEX complements existing databases and tools such as ATTED-II, CoP and STARNET 2.

## Availability and requirements

**Project name:** PLANEX

**Operating system(s):** Platform Independent (tested on Windows, i386 Linux and Mac)

**Programming Languages:** Perl

**Other requirements:** Web browser (tested on Chrome, Safari and Explorer)

**License:** Creative Commons Attribution License

**The serve is freely available at**http://planex.plantbioinformatics.org

## Abbreviations

ACT: Arabidopsis co-expression toolkit; AIX: Advanced interactive eXecutive; DFCI: Dana faber cancer institute; GEO: Gene expression omnibus; GO: Gene ontology; KEGG: Kyoto encyclopedia of genes and genomes; PCA: Principal components analysis; PCC: Pearson’s correlation coefficients; PLANEX: The PLAnt co-EXpression database; PPIs: Protein-protein interactions; SGD: *Saccharomyces* genome database; TAIR: The arabidopsis information resource.

## Competing interests

The authors declare that they have no competing interests**.**

## Authors’ contributions

WCY, YY and CSJ designed and implemented the database. WCY and YY constructed website pages and on-line tools. WCY, CSJ, KS and BML were responsible for data collection. WCY and KS participated in the design of the database schema. WCY and BML conceived the study. WCY, CSJ, KS and BML drafted the manuscript. All authors read and approved the manuscript.

## Supplementary Material

Additional file 1: Table S1 Public microarray data information in PLANEX. Click here for file

## References

[B1] Eisen MB Spellman PT Brown PO Botstein D Cluster analysis and display of genome-wide expression patterns Proc Natl Acad Sci U S A 1998 95 14863 14868 10.1073/pnas.95.25.14863 9843981 PMC24541

[B2] Lee HK Hsu AK Sajdak J Qin J Pavlidis P Coexpression analysis of human genes across many microarray data sets Genome Res 2004 14 1085 1094 10.1101/gr.1910904 15173114 PMC419787

[B3] Aoki K Ogata Y Shibata D Approaches for extracting practical information from gene co-expression networks in plant biology Plant Cell Physiol 2007 48 381 390 10.1093/pcp/pcm013 17251202

[B4] Brazma A Parkinson H Sarkans U Shojatalab M Vilo J Abeygunawardena N Holloway E Kapushesky M Kemmeren P Lara GG ArrayExpress a public repository for microarray gene expression data at the EBI Nucl Acids Res 2003 31 68 71 10.1093/nar/gkg091 12519949 PMC165538

[B5] Barrett T Troup DB Wilhite SE Ledoux P Rudnev D Evangelista C Kim IF Soboleva A Tomashevsky M Edgar R NCBI GEO: mining tens of millions of expression profiles–database and tools update Nucl Acids Res 2007 35 D760 D765 10.1093/nar/gkl887 17099226 PMC1669752

[B6] Craigon DJ James N Okyere J Higgins J Jotham J May S NASCArrays: a repository for microarray data generated by NASC’s transcriptomics service Nucl Acids Res 2004 32 D575 D577 10.1093/nar/gkh133 14681484 PMC308867

[B7] Zimmermann P Hirsch-Hoffmann M Hennig L Gruissem W GENEVESTIGATOR. Arabidopsis microarray database and analysis toolbox Plant Physiol 2004 36 2621 2632 10.1104/pp.104.046367 PMC523327 15375207

[B8] Analysis of the genome sequence of the flowering plant Arabidopsis thaliana Nature 2000 408 796 815 10.1038/35048692 11130711

[B9] Schmutz J Cannon SB Schlueter J Ma J Mitros T Nelson W Hyten DL Song Q Thelen JJ Cheng J Genome sequence of the palaeopolyploid soybean Nature 2010 463 178 183 10.1038/nature08670 20075913

[B10] Ouyang S Zhu W Hamilton J Lin H Campbell M Childs K Thibaud-Nissen F Malek RL Lee Y Zheng L The TIGR rice genome annotation resource: improvements and new features Nucl Acids Res 2007 35 D883 D887 10.1093/nar/gkl976 17145706 PMC1751532

[B11] Tomato Genome Consortium The tomato genome sequence provides insights into fleshy fruit evolution Nature 2012 485 635 664 10.1038/nature11119 22660326 PMC3378239

[B12] Jaillon O Aury J-M Noel B Policriti A Clepet C Casagrande A Choisne N Aubourg S Vitulo N Jubin C Vezzi A Legeai F Hugueney P Dasilva C Horner D Mica E Jublot D Poulain J Bruyère C Billault A Segurens B Gouyvenoux M Ugarte E Cattonaro F Anthouard V Vico V Del Fabbro C Alaux M Di Gaspero G Dumas V The grapevine genome sequence suggests ancestral hexaploidization in major angiosperm phyla Nature 2007 449 463 467 10.1038/nature06148 17721507

[B13] Schnable PS Ware D Fulton RS Stein JC Wei F Pasternak S Liang C Zhang J Fulton L Graves TA The B73 maize genome: complexity, diversity, and dynamics Science 2009 326 1112 1115 10.1126/science.1178534 19965430

[B14] Antonescu C Antonescu V Sultana R Quackenbush J Using the DFCI gene index databases for biological discovery Curr Protoc Bioinformatics 2010 Chapter 1:Unit1.6.1-36 10.1002/0471250953.bi0106s29 PMC4205239 20205187

[B15] Irizarry RA Hobbs B Collin F Beazer-Barclay YD Antonellis KJ Scherf U Speed TP Exploration, normalization, and summaries of high density oligonucleotide array probe level data Biostatistics 2003 4 249 264 10.1093/biostatistics/4.2.249 12925520

[B16] Wu Z Irizarry R Gentleman R Martinez-Murillo F Spencer F A model-based background adjustment for oligonucleotide expression arrays J Am Stat Assoc 2004 99 909 917 10.1198/016214504000000683

[B17] Hubbell E Liu WM Mei R Robust estimators for expression analysis Bioinformatics 2002 18 1585 1592 10.1093/bioinformatics/18.12.1585 12490442

[B18] Soper HE Young AE Cave BM Lee A Pearson K On the distribution of the correlation coefficient in small samples. appendixAppendix ii to the papers of “student” and r. a. Fisher. a cooperative study Biometrika 1917 11 328 413 10.1093/biomet/11.4.328

[B19] Brunet JP Tamayo P Golub TR Mesirov JP Metagenes and molecular pattern discovery using matrix factorization Proc Natl Acad Sci USA 2004 101 4164 41693 10.1073/pnas.0308531101 15016911 PMC384712

[B20] Manfield IW Jen CH Pinney JW Michalopoulos I Bradford JR Gilmartin PM Westhead DR Arabidopsis co-expression tool (ACT): web server tools for microarray-based gene expression analysis Nucl Acids Res 2006 34 W504 W509 10.1093/nar/gkl204 16845059 PMC1538833

[B21] Jupiter D Chen H Van Buren V STARNET 2: a web-based tool for accelerating discovery of gene regulatory networks using microarray co-expression data BMC Bioinforma 2009 10 332 10.1186/1471-2105-10-332 PMC2765977 19828039

[B22] Lee TH Kim YK Pham TTM Song SI Kim JK Kang KY An G Jung KH Galbraith DW Kim M RiceArrayNet: a database for correlating gene expression from transcriptome profiling, and its application to the analysis of coexpressed genes in rice Plant Physiol 2009 151 16 33 10.1104/pp.109.139030 19605550 PMC2735985

[B23] Obayashi T Hayashi S Saeki M Ohta H Kinoshita K ATTED-II provides coexpressed gene networks for Arabidopsis Nucleic Acids Res 2009 37 D987 D991 10.1093/nar/gkn807 18953027 PMC2686564

[B24] Ogata Y Suzuki H Sakurai N Shibata D CoP: a database for characterizing co-expressed gene modules with biological information in plants Bioinformatics 2010 26 1267 1268 10.1093/bioinformatics/btq121 20305269

[B25] Mutwil M Klie S Tohge T Giorgi FM Wilkins O Campbell MM Fernie AR Usadel B Nikoloski Z Persson S PlaNet: combined sequence and expression comparisons across plant networks derived from seven species Plant Cell 2011 23 895 910 10.1105/tpc.111.083667 21441431 PMC3082271

[B26] Affymetrixhttp://www.affymetrix.com/estore/

[B27] KISTI Super Computing Centerhttp://www.ksc.re.kr/

[B28] Staff Using MPI-portable parallel programming with the message-passing interface William Gropp Sci Program 1996 5 275 276

[B29] Hartigan JA Wong MA Algorithm AS 136: a K-means clustering algorithm J Royal Stat Soc Series C (Applied Statistics) 1979 28 100 108

[B30] Parallel K-Means Data Clustering http://users.eecs.northwestern.edu/~wkliao/Kmeans/

[B31] Phytozomehttp://www.phytozome.net

[B32] Hunter S Apweiler R Attwood TK Bairoch A Bateman A Binns D Bork P Das U Daugherty L Duquenne L Finn RD Gough J Haft D Hulo N Kahn D Kelly E Laugraud A Letunic I Lonsdale D Lopez R Madera M Maslen J McAnulla C McDowall J Mistry J Mitchell A Mulder N Natale D Orengo C Quinn AF InterPro: the integrative protein signature database Nucleic Acids Res 2009 37 D211 D215 10.1093/nar/gkn785 18940856 PMC2686546

[B33] Boyle EI Weng S Gollub J Jin H Botstein D Cherry JM Sherlock G GO: TermFinder–open source software for accessing gene ontology information and finding significantly enriched gene ontology terms associated with a list of genes Bioinformatics 2004 20 3710 3715 10.1093/bioinformatics/bth456 15297299 PMC3037731

[B34] Cohen J A coefficient of agreement for nominal scales Educ Psychol Meas 1960 20 37 46 10.1177/001316446002000104

[B35] Huang DW Sherman BT Lempicki RA Systematic and integrative analysis of large gene lists using DAVID bioinformatics resources Nat Protoc 2009 4 44 57 19131956 10.1038/nprot.2008.211

[B36] Huang DW Sherman BT Lempicki RA Bioinformatics enrichment tools: paths toward the comprehensive functional analysis of large gene lists Nucleic Acids Res 2009 37 1 13 10.1093/nar/gkn923 19033363 PMC2615629

[B37] Bateman A Coin L Durbin R Finn RD Hollich V Griffiths-Jones S Khanna A Marshall M Moxon S Sonnhammer ELL Studholme DJ Yeats C Eddy SR The Pfam protein families database Nucleic Acids Res 2004 32 D138 D141 10.1093/nar/gkh121 14681378 PMC308855

[B38] Dancerhttp://www.perldancer.org

[B39] Jqueryhttp://www.jquery.com

[B40] MongoDBhttp://www.mongodb.com

[B41] Tokyocabinethttp://fallabs.com/tokyocabinet

[B42] Lopes CT Franz M Kazi F Donaldson SL Morris Q Bader GD Cytoscape Web: an interactive web-based network browser Bioinformatics 2010 26 2347 2348 10.1093/bioinformatics/btq430 20656902 PMC2935447

[B43] Ubuntuhttp://www.ubuntu.com

[B44] Johnson M Zaretskaya I Raytselis Y Merezhuk Y McGinnis S Madden TL NCBI BLAST: a better web interface Nucleic Acids Res 2008 36 W5 W9 10.1093/nar/gkn201 18440982 PMC2447716

[B45] Yim WC Lee B-M Jang CS Expression diversity and evolutionary dynamics of rice duplicate genes Mol Genet Genomics 2009 281 483 493 10.1007/s00438-009-0425-y 19184107

[B46] Aoki K Ogata Y Shibata D Approaches for extracting practical information from gene co-expression networks in plant biology Plant Cell Physiol 2007 48 381 390 10.1093/pcp/pcm013 17251202

[B47] Simillion C Vandepoele K Van Montagu MCE Zabeau M Van de Peer Y The hidden duplication past of Arabidopsis thaliana Proc Natl Acad Sci USA 2002 99 13627 13632 10.1073/pnas.212522399 12374856 PMC129725

[B48] Bowers JE Chapman BA Rong J Paterson AH Unravelling angiosperm genome evolution by phylogenetic analysis of chromosomal duplication events Nature 2003 422 433 438 10.1038/nature01521 12660784

[B49] Jang CS Yim WC Moon J-C Jung JH Lee TG Lim SD Cho SH Lee KK Kim W Seo YW Lee B-M Evolution of non-specific lipid transfer protein (nsLTP) genes in the Poaceae family: their duplication and diversity Mol Genet Genomics 2008 279 481 497 10.1007/s00438-008-0327-4 18270740

